# Mission possible: An attempt toward a phylogenetic understanding of the genus *Dendrobaena* (Crassiclitellata, Lumbricidae)

**DOI:** 10.3897/zookeys.1286.201330

**Published:** 2026-07-24

**Authors:** Tímea Szederjesi, Tomáš Pavlíček, Angelika Kliszcz, Csaba Csuzdi

**Affiliations:** 1 Department of Zoology, Eszterházy Károly Catholic University, 3300 Eger, 12D Leányka str., Hungary Department of Zoology, Eszterházy Károly Catholic University Eger Hungary; 2 Chafé (Viana do Castelo), Portugal Department of Agroecology and Plant Production, University of Agriculture in Krakow Krakow Poland; 3 Department of Agroecology and Plant Production, University of Agriculture in Krakow, Krakow, Poland Unaffiliated Chafé Portugal; 4 39 Kenderesi str., Piliscsaba, Hungary Unaffiliated Piliscsaba Hungary

**Keywords:** Earthworms, Oligochaeta, phylogenetics, polyphyly, taxonomy

## Abstract

With approximately 115 described species, *Dendrobaena* is the most species-rich genus within the family Lumbricidae and is probably also the most heterogeneous, both morphologically and geographically. Early attempts at morphological revision led to the separation of some morphologically homogeneous groups as separate genera, such as *Fitzingeria* and *Satchellius*. Although molecular phylogenetic approaches are now widely applied, relatively few studies have focused on *Dendrobaena*, and those have generally been limited to the revision of individual species groups. To address this gap, we employed an integrative approach combining molecular and morphological data. Our dataset includes 40 *Dendrobaena* taxa from diverse regions of Europe and Asia, several *Eisenia* species representing their entire geographic range, and representatives of related genera (*Dendrodriloides*, *Healyella*, and *Spermophorodrilus*). Our results indicate the potential polyphyly of both *Dendrobaena* and *Eisenia*, with *E. ebneri* and related species forming a clade with Caucasian *Dendrodriloides* species instead of *Eisenia* s. str. This clade also includes *Dendrobaena veneta* and closely related taxa. Within *Dendrobaena*, five distinct clades were recovered. According to our results, the lineage comprising former *Fitzingeria* species and allied taxa, and also the *D. byblica* species group together with *Philomontanus baloutchi*, do not belong to the *Dendrobaena* s. str. clade.

## Introduction

The earthworm genus *Dendrobaena* Omodeo, 1956 is the most species-rich genus within the family Lumbricidae, comprising approximately 115 species (Csuzdi 2012). At the same time, it is one of the most heterogeneous genera with respect to both morphological characteristics and geographic distribution. Considerable interspecific variation occurs in the structure of the longitudinal musculature (with both pinnate and fasciculate types present), the morphology of the calciferous glands (varying in degree of development and in the presence or absence of diverticula), the position of the last pair of hearts (located in segments 9, 10, or 11 and sometimes with or without extraoesophageal vessels), pigmentation (ranging from dark purple-red to completely unpigmented), and setal arrangement (from widely spaced to closely paired setae). This pronounced heterogeneity has long necessitated the need for a comprehensive taxonomic revision of the genus (Csuzdi and Zicsi 2003).

In his morphology-based revision, Csuzdi (1984) differentiated seven main species groups in the classical *Dendrobaena* (sensu Pop 1941); the *platyura* group, *osellai* group, *schmidti* group, *veneta* group, *octaedra* group, *mammalis* group, and *byblica* group. The *platyura* group is characterized by a posterior displacement of the male pores (located on segments 25 or 26 instead of segment 15) and was therefore separated as the genus *Fitzingeria* by Zicsi (1978). However, based on an integrated taxonomic revision, the group was subsequently reassigned to *Dendrobaena* by Szederjesi et al. (2018), as the posterior shift of the male pores was shown to represent a homoplasy rather than homology.

The *osellai* group is characterized by fasciculate longitudinal musculature and bilobate nephridial bladders. Its representatives were transferred to the genus *Kritodrilus* Bouché, 1972 by Omodeo and Rota (1989) and later also by Zicsi and Csuzdi (1999). However, recent molecular studies do not support this taxonomic placement. Further DNA-based analyses are therefore required to determine whether this group represents an independent genus (Marchán et al. 2021a; Pinadero et al. 2023).

Another well-characterized group comprises *D. mammalis* and related species, which have large calciferous diverticula in segment 10 and the posterior pair of hearts located in segment 11, with extraoesophageal vessels in segment 12. These species are currently placed in a separate genus *Satchellius* Gates, 1975 (Szederjesi and Csuzdi 2016).

*Dendrobaena schmidti* (Michaelsen, 1907) and some similar species form a separate group due to their well-developed calciferous diverticula in segment 12 and the presence of an extraoesophageal vessel in segment 12. Therefore, Kvavadze (2000a) created the subgenus *Caucasodrilus* within *Dendrobaena* to accommodate them.

*Dendrobaena byblica* (Rosa, 1893) and its related species, which lack calciferous diverticula and, in most cases, dark-red pigmentation, form another distinct group within the genus *Dendrobaena*, as also corroborated by the integrated taxonomic revision of Szederjesi et al. (2018). Members of this group are distributed across the Mediterranean region and Central Asia. For this group, Kvavadze (1993) established the genus *Omodeoia*, diagnosed by the following characters: unpaired setae; tristriated genital setae; clitellum terminating on segment 30; three or four pairs of seminal vesicles; spermathecal ducts opening above setal line *d* or on *d*, rarely on *c*; and a basal chromosome number of *n* = 17. However, this genus has not been widely accepted by earthworm taxonomists.

In case of the type species of the genus, *D. octaedra* (Savigny, 1826) and its relatives, the last pair of hearts is usually found in segment 9 or 10, and if in 11, then it is less developed. The nephridial bladders are typically biscuit- or sausage-shaped and the calciferous glands are well developed usually in both 11 and 12.

*Dendrobaena veneta* (Rosa, 1886) and its close relatives form a distinct group within *Dendrobaena*, characterized by fasciculate longitudinal musculature and underdeveloped calciferous glands lacking diverticula. Some species (e.g. *D. hrabei* (Černosvitov, 1934)) exhibit a more closely spaced setal arrangement, which is not a typical feature of the genus *Dendrobaena*. On this basis, Csuzdi (1984) suggested that the *D. veneta* group may represent a transitional lineage towards the genus *Eisenia* Malm, 1887 and may be related to *E. kattoulasi* Zicsi & Michalis, 1981. He further noted that, based on these shared characters, *Eisenia grandis* Michaelsen, 1907 and its related species should also be included in the *D. veneta* group, and that this species group may warrant recognition as a separate genus.

Kvavadze (2000b) established the genus *Dendrodriloides* for the former *E. grandis* and related species distributed in the Caucasus region and northeastern Anatolia. These species are peculiar with their tetrahedral genital setae, spermathecal openings near the mid-dorsal line and closely paired setae. Kvavadze suggested that some other Southern Balkanic *Eisenia* species (*E. ebneri* (Michaelsen, 1914), *E. storkani* (Černosvitov, 1934), *E. kattoulasi*) and the Anatolian *D. montana* (Michaelsen, 1910) should also be included in this new genus. Szederjesi and Csuzdi (2015) showed that the above mentioned Balkanic *Eisenia* species, together with *E. oreophila* Szederjesi & Csuzdi, 2012, indeed have tetrahedral genital setae and based on the COI sequences, form a well-separated group in the genus *Eisenia*.

Since the comprehensive study of Csuzdi (1984), little effort has been made to revise the genus *Dendrobaena* as a whole. One of the first molecular phylogenetic studies was the pioneer work of Cech et al. (2006), who used the 18S rDNA sequences to reveal the phylogenetic relationships within the genus and between some closely related genera. They showed the polyphyletic nature of the genus *Bimastos* Moore, 1893 for the first time, of which several earlier species were put into *Dendrobaena* by Zicsi (1981), and they confirmed the validity of the genus *Healyella* Omodeo & Rota, 1989. This work also revealed the distinctness of *D. cognettii* (Michaelsen, 1903), which stands far from the other *Dendrobaena* s.l. species. Since then, most works have focused on smaller species groups, e.g. *D. alpina* (Rosa, 1884), *D. byblica*, *D. veneta* (Csuzdi et al. 2005, 2023; Szederjesi et al. 2018, 2019, 2025), and *D. illyrica* (Cognetti, 1906) (Popović et al. 2025).

In a recent larger-scale study, Shekhovtsov et al. (2024) used complete or nearly complete mitochondrial genomes to reveal the relationships within the family Lumbricidae, with special attention to *Dendrobaena*. Their study clearly showed that the *D. byblica* species group is far from the other *Dendrobaena* species and form a well-separated clade. Another interesting result is that the species *Dd. grandis* is nested among *Dendrobaena*, and stands closest to the *D. veneta*–*D. hortensis* (Michaelsen, 1890) clade. Unfortunately, this study included only a relatively small number of *Dendrobaena* species with limited geographic range.

As this overview demonstrates, no comprehensive taxonomic revision has yet studied a large number of *Dendrobaena* species spanning most of the geographic range of the genus. The present integrative taxonomic study aims to fill this gap by including 40 taxa of the genus *Dendrobaena* from Central Europe, the Balkans, Anatolia, the Middle East, the Caucasus, and Central Asia, along with *Eisenia* species from across its full range and also representatives of related genera (*Dendrodriloides*, *Healyella*, and *Spermophorodrilus*).

## Material and methods

### DNA isolation, amplification and sequencing

Molecular studies were carried out at the Molecular Taxonomy Laboratory of the Hungarian Natural History Museum, Budapest. Parts of the muscular body wall behind the clitellum were cut and cleaned for analysis. DNA extraction was performed with QIAamp DNA Micro Kit (Qiagen) following the manufacturer’s protocol. The mitochondrial COI, 16S rDNA and the nuclear ITS2 regions were used in this study. The primers HCO 2198 (5'-TAAACT TCA GGG TGA CCA AAA AAT CA-3') and LCO 1490 (5'-GGT CAA CAA ATC ATA AAG ATA TTG G-3') were used for the amplification of the COI sequences (Folmer et al. 1994). The PCR mix (total volume 25 μl) consisted of 100 ng DNA template, 1× Dream Taq Buffer, 0.5 mM of MgCl_2_, 0.25 mM of dNTPs, 35 μg of bovineserum albumin, 0.3 μM of each primer, 0.5 units of Dream Taq Polymerase (Thermo Scientific). For amplification of the 16S rDNA sequences, the primers 16 sar (5'-CGC CTG TTT ATC AAA AAC AT-3') and 16 sbr (5'-CCGGTY TGA ACT CAG ATC AYG T-3') were used (Palumbi et al. 1991). The PCR mix was of identical composition and volume as with COI. The partial 5.8 rDNA-ITS2-partial 28S rDNA (ITS2) sequences were amplified with the use of the primers R5.8S1 (5'-CGA TGA AGA GCG CAG CCA GC-3') and BD2 (5'-TAT GCT TAA ATT CAGCGG GT-3') (Perrot-Minnot 2004; Chen et al. 2002). The PCR mix (total volume 50 μl) consisted of 100 ng DNA template, 1× Dream Taq Buffer, 1.5 mM of MgCl2, 0.2 mM of dNTPs, 5% of DMSO, 1.25 μM of each primer, 0.5 units of Dream Taq Polymerase (Thermo Scientific). The PCR product was purified using High Pure PCRProduct Purification Kit (Roche). The sequencing reactions were performed with BigDye Terminator v. 3.1 Cycle Sequencing Kit. After the removal of the unincorporated reaction components using BigDye XTerminator Kit, sequences were run on an ABI 3130 genetic analyzer. Newly obtained sequences were uploaded to the GenBank nucleotide database (Benson et al. 2012; https://ncbi.nlm.nih.gov/Genbank).

### Phylogenetic reconstruction and visualisation

Altogether 122 specimens’ sequences were used in this study (Suppl. material 1). A part of the sequences was downloaded from the GenBank nucleotide database, newly obtained sequences are marked in bold. **HNHM** refers to the earthworm collection of the Hungarian Natural History Museum.

Besides the genera in focus, representatives of several other lumbricid genera were included in the study: *Bimastos*, *Aporrectodea*, *Octolasion*, *Helodrilus*, *Imetescolex*, *Proctodrilus*, and *Cernosvitovia*.

The alignment was performed with MAFFT v. 7 (Katoh and Standley 2013) using the E-INS-i option settings and concatenated with MEGA 11 (Tamura et al. 2021), resulting in a matrix of 1,662 bp (COI = 658 bp, 16S = 521 bp, 5.8S-ITS2 = 481 bp). The 5.8S-ITS2 sequences were gblock optimised (Castresana 2000) allowing gap positions in the final blocks as implemented in the Phylogeny.fr website (Dereeper et al. 2008).

The best-fitting evolutionary model for each partition was selected using ModelFinder (Kalyaanamoorthy et al. 2017) implemented in the IQTree v. 3.1.1 (Nguyen et al. 2015) by applying the Akaike information criterion (AIC; Akaike 1973) and the Bayesian information criterion (BIC; Schwarz 1978). For all the three partitions the best fit model was GTR + I + G4 + F.

For the Bayesian inference the GTR + I + G model was used and the phylogeny was estimated with MrBayes v. 3.2.7a (Ronquist et al. 2012). Each of the two independent runs was set to 10 million generations sampling a tree every 1,000 generations. After removing the first 2,000 trees as burn-in, the remaining trees were combined and summarized on a 50% majority-rule consensus tree. The maximum-likelihood analysis was conducted using IQTree v. 3.1.1 on the PhyloSuite desktop platform (Zhang et al. 2020) with 1,000 bootstrap repeats. FigTree v. 1.4.4 (https://tree.bio.ed.ac.uk/software/figtree/) was used for visualization. Only the bootstrap values above 75% and posterior probabilities above 0.75 are shown on the figures.

Distribution maps were created with QGIS v. 3.44.8 (https://qgis.org/).

## Results

**Figure 1.**
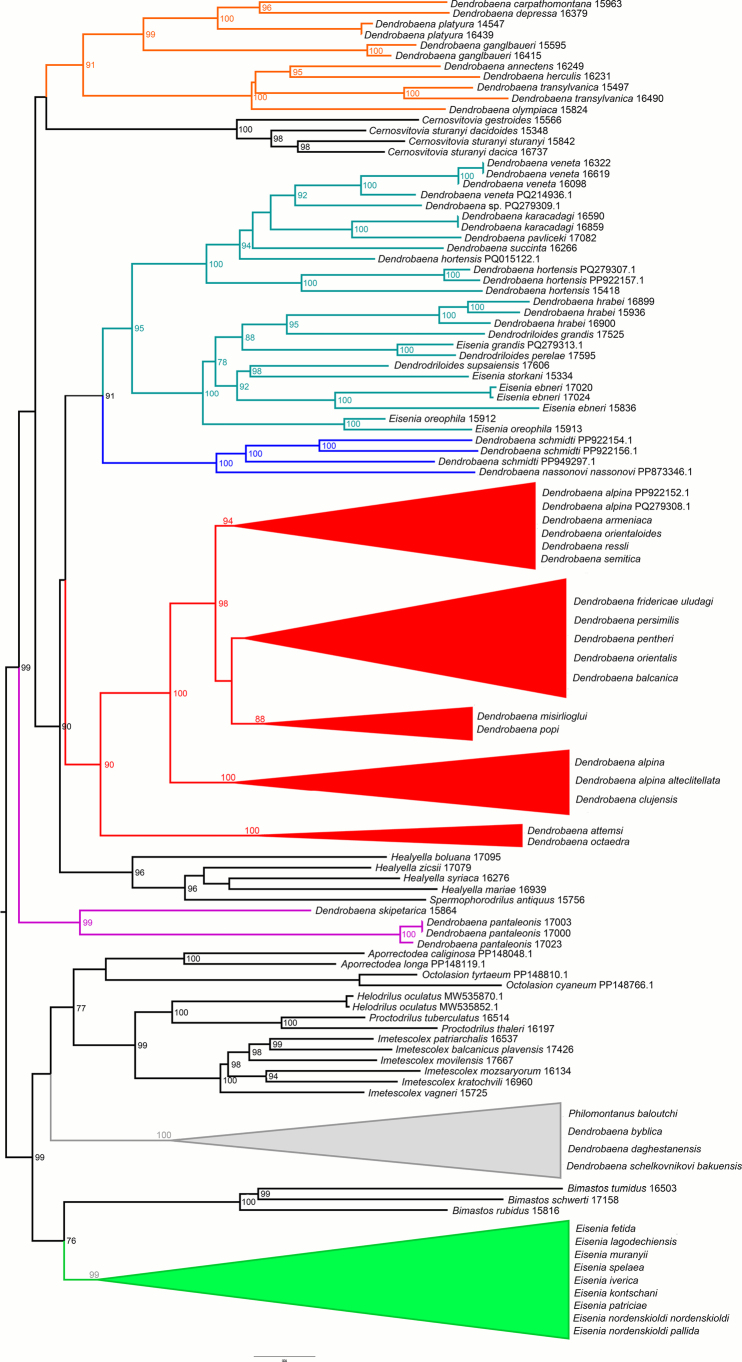
Maximum-likelihood tree obtained from the phylogenetic analysis of the concatenated sequences of the molecular markers COI, 16S rDNA, and ITS2. Numbers indicate bootstrap supports. Explanation of the colours is in the text.

**Figure 2.**
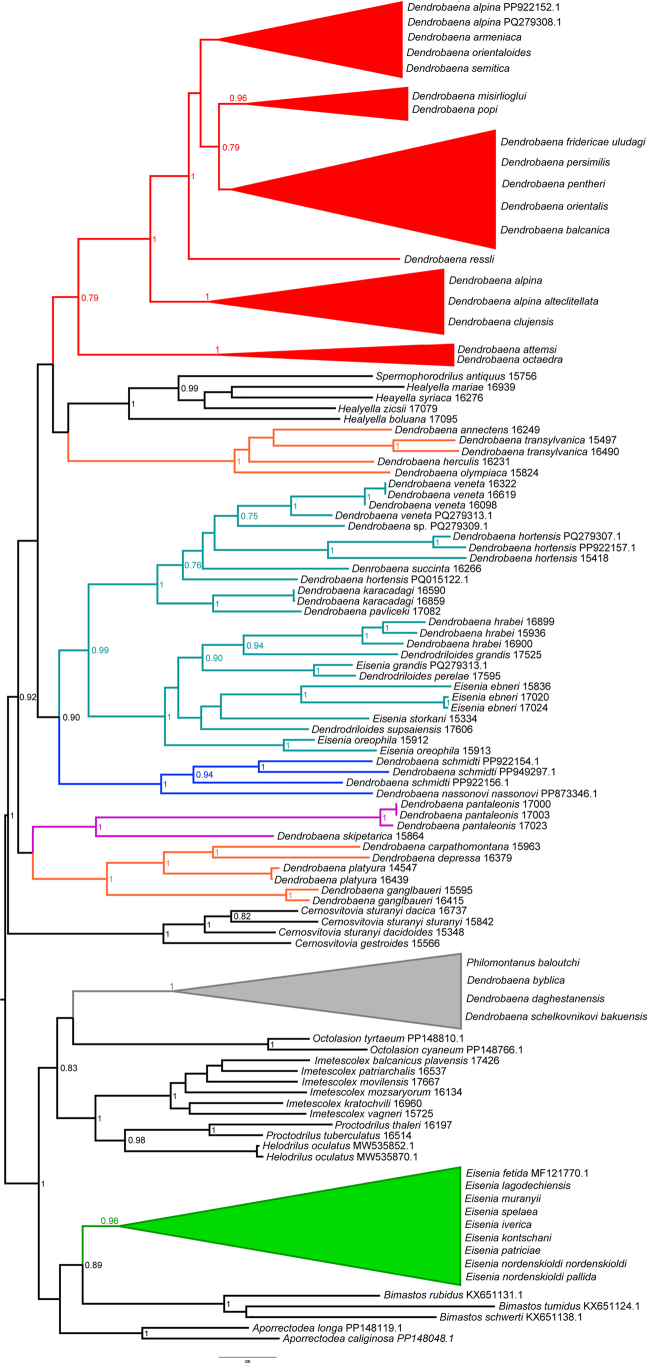
Bayesian inference tree built by the analysis of the concatenated sequences of the molecular markers COI, 16S rDNA, and ITS2. Numbers indicate posterior probabilities. Explanation of the colours is in the text.

Our results (Figs 1, 2) suggest the possible polyphyly of both the *Eisenia* and *Dendrobaena* genera. *Eisenia fetida* (Savigny, 1826), the type species of the genus, groups together with other Central European, Balkanic, Caucasian, Upper Mesopotamian, and Asian *Eisenia* species (marked with green) (99% bootstrap support and 0.96 posterior probability). The Southern Balkanic *E. ebneri* species group containing *E. ebneri*, *E. storkani*, and *E. oreophila* is placed outside of *Eisenia* s. str. and forms a clade together with the Caucasian *Dendrodriloides* species and *D. hrabei* (100% bootstrap, 1 pp). Their sister clade (95% bootstrap, 099 pp) includes *D. veneta*, *D. succinta* (Rosa, 1905), *D. hortensis* (Michaelsen, 1890), *D. pavliceki* Szederjesi & Csuzdi, 2018, *D. karacadagi* Szederjesi, Pavlíček & Csuzdi, 2019, and an unidentified *Dendrobaena* species from Azerbaijan (isolate E4768 in Shekhovtsov et al. 2024) (100% bootstrap, 1 pp) (marked in turquoise). The Caucasian *Dendrobaena schmidti* and its close ally, *D. nassonovi* Kulagin, 1889 are sister to this branch (91% bootstrap, 0.90 pp) (in dark blue).

**Figure 3.**
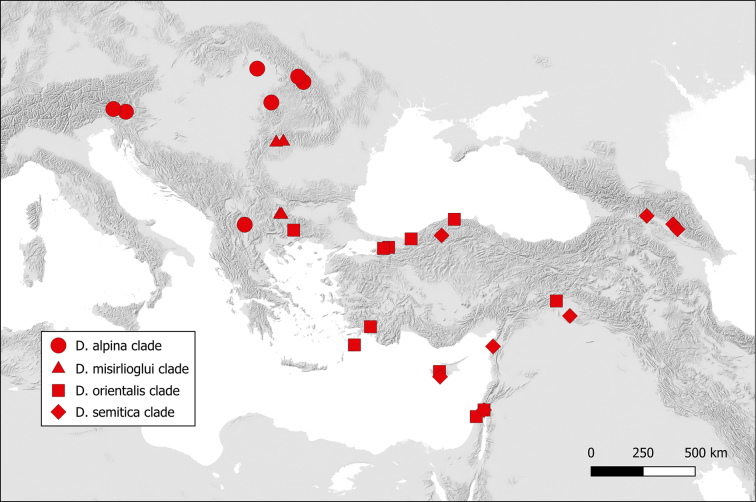
Collecting localities of the specimens of the *Dendrobaena* s. str. (red) clade, subclades are displayed based on the ML tree. Widely distributed species are not included.

*Dendrobaena* sensu stricto (90% bootstrap) is resolved into five clades in our analysis (shown in red). The type species, *D. octaedra*, occupies an early-branching position together with *D. attemsi* (Michaelsen, 1902) (100% bootstrap, 1 pp). The Alpine–Carpathian–Balkanic (100% bootstrap, 1 pp) and Balkanic–Anatolian–Levantine groups (98% bootstrap, 1 pp) identified by Csuzdi et al. (2023) and Szederjesi et al. (2025) are clearly recovered in our study, with the Balkanic–Anatolian–Levantine group further subdivided into three distinct subgroups (Fig. 3).

**Figure 4.**
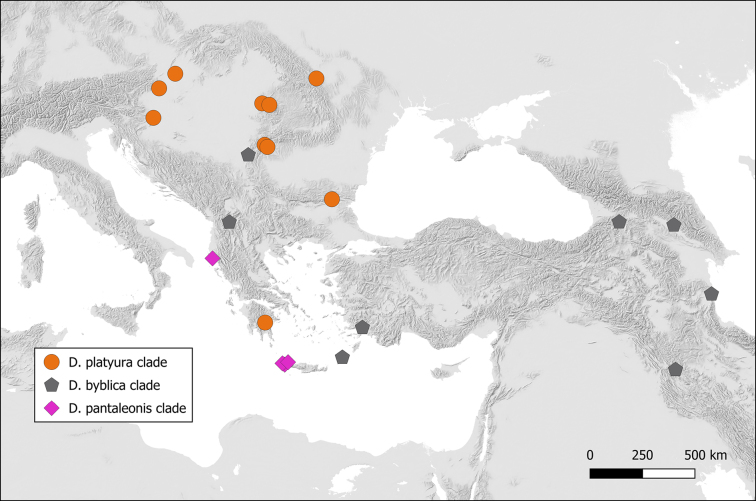
Collecting localities of the specimens of the *D. platyura* "*Fitzingeria*” (orange) clade, *D. byblica* "*Omodeoia*” (grey) clade, and the *D. pantaleonis* (purple) clade.

The peculiar *D. pantaleonis* (Chinaglia, 1913) and *D. skipetarica* Szederjesi & Csuzdi, 2017 (in purple) (99% bootstrap, 1 pp) fall outside the *Dendrobaena* sensu stricto clade, as does the clade formed by the former *Fitzingeria* species (*platyura*, *depressa*, *carpathomontana*, *annectens*) together with the Romanian *D. herculis* Szederjesi, Pop & Csuzdi, 2017 and *D. transylvanica* Szederjesi, Pop & Csuzdi, 2017, the Southern Balkanic *D. olympiaca* (Michaelsen, 1902), and the Illyrian *D. ganglbaueri* (Rosa, 1894) (shown in orange) (91% bootstrap) (Fig. 4). However, this latter clade has not been recovered as monophyletic on the tree obtained from the Bayesian inference (Fig. 2) and instead split into two subclades, with low support.

The *D. byblica* species group is also placed outside *Dendrobaena* (in grey) (100% bootstrap, 1 pp). This clade includes the recently described genus *Philomontanus*, which is represented by *Ph. baloutchi* Bozorgi & Malek, 2019.

## Discussion

The separation of the Southern Balkanic *E. ebneri* species group from *Eisenia* sensu stricto is not surprising, as the distinctness of the two groups has already been demonstrated by both morphological and molecular phylogenetic studies (Szederjesi and Csuzdi 2015). Whereas *Eisenia* s. str. species possess trihedral genital setae, members of the *E. ebneri* group have tetrahedral genital setae, a condition also observed in the Caucasian *Dendrodriloides* species represented here by *Dd. grandis*, *Dd. perelae* (Kvavadze, 1973), and *Dd. supsaiensis* (Kvavadze, 1985).

Our study is the first to demonstrate a close molecular phylogenetic relationship between the *E. ebneri* and *Dd. grandis* species groups. However, a close relationship based on morphological characters had already been proposed by Zicsi and Michalis (1981) and Mršić (1991), who treated *ebneri* and *storkani* as subspecies of *grandis* within the genus *Eisenia*. *Dendrobaena hrabei* also joins this assemblage; this species is unusual among *Dendrobaena* in having closely paired setae.

The sister clade of these species is the *D. veneta* species group. A close phylogenetic relationship between *D. veneta* and *Dd. grandis* has already been suggested by Zicsi and Csuzdi (1986) and Csuzdi and Zicsi (2003) on morphological evidences and was also supported by Shekhovtsov et al. (2024) using complete mitochondrial genomes; however, in the latter study all these taxa were nested within *Dendrobaena*. The species conglomerate highlighted in turquoise in Figs 1, 2 shares several characteristic morphological features, including fasciculate longitudinal musculature, less developed calciferous glands lacking diverticula, and a broadly similar position of the clitellum (approximately segments 26–33) and tubercles (approximately segments 30–31).

With respect to setal arrangement, a gradual transition can be observed from distantly spaced setae (e.g. *D. veneta*), through an intermediate condition with more closely spaced setae (*D. hrabei*), to closely paired setae (e.g. *E. oreophila*). Pigmentation is likewise variable, ranging from dark red-violet to completely unpigmented species. The structure of the genital setae has been examined in only a few taxa and therefore requires further investigation.

**Figure 5.**
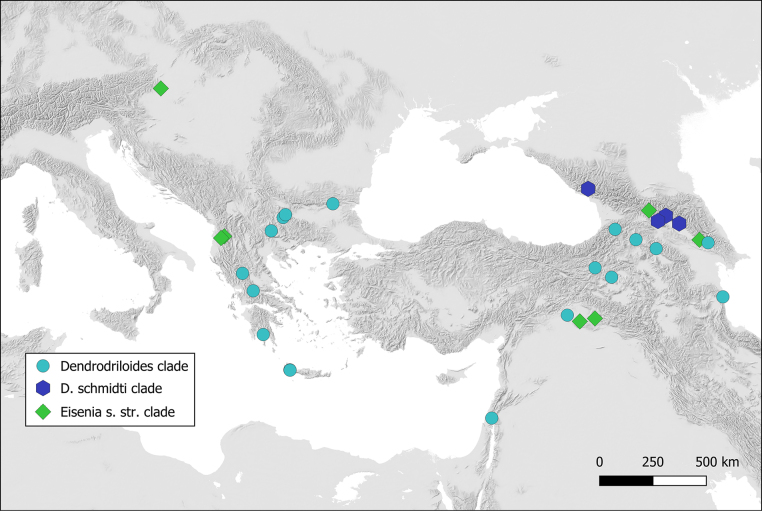
Collecting localities of the specimens of the "*Dendrodriloides*” (turquise) clade, *D. schmidti* clade (blue), and the *Eisenia* sensu stricto clade (green). Widely distributed species are not included.

This large clade shows an Anatolian–Levantine–Southern Balkanic distribution (Fig. 5), which is likely linked to the dynamic and complex geological history of the Eastern Mediterranean. As a result of the collision of the African and Eurasian plates, and the gradual northward movement of the African and Arabian plates, the Aegeid land mass formed during the Oligocene epoch (Rögl 1999) providing biogeographical connection between the present-day Balkan and Anatolia (Balkanatolia sensu Licht et al. 2022). In the Miocene, this land mass had dinamic and changing land connections, until the formation of the Aegean Sea divided it to a northern and southern part (Poulakakis et al. 2015). The southernmost part of the Balkan Peninsula, the Cyclades, and Crete were formed from the Northern Aegeid, while Anatolia, Levante, and the surrounding islands were formed from the Southern Aegeid (Rögl 1999). Following this period, the Northern and Southern Aegeids were reconnected during the Messinean Salinity Crisis (MSCP, 5.96–5.33 Mya) (Krijgsman et al. 1999) and during the Quaternary Ice Age (Casale and Taglianti 1999).

Based on the results of the molecular phylogenetic analyses, the morphological features, and the distribution as well, this large clade may represent a separate genus. However, further investigations, including genomic approaches and more detailed morphological studies, especially the shape of the genical setae, are needed to corroborate this hypothesis.

Another distinct clade found outside *Dendrobaena* sensu stricto is formed by *D. schmidti* and *D. nassonovi*. These Caucasian species are characterized by pinnate longitudinal musculature, well-defined calciferous glands in segments 11 and 12, usually with diverticula in segment 12, spermathecal openings in or below setal line *d*, and a clitellum that usually terminates on segment 32.

Several species formerly assigned to the genus *Fitzingeria*, together with *D. ganglbaueri*, *D. olympiaca*, and some recently described taxa, are also recovered outside *Dendrobaena* sensu stricto. The ML analysis supported the monophyly of this clade marked with orange however, it split into two low supported subclades on the Bayesian tree. The synapomorphies of this whole group include pinnate longitudinal musculature, the presence of calciferous diverticula in segment 11, and dark red pigmentation (Szederjesi et al. 2018). The position of the male pore is variable within this assemblage, occurring both in the typical position on segment 15 and in a posterior position on segments 26 or 27. These species are distributed across Central Europe and the Balkans. As this group of species is uniform regarding several morphological features and their distribution as well, the result of the Bayesian inference that splitted this clade into two separate sublades might be due to the small number of markers used. Thus, genomic studies are needed to clarify the relationships of these species.

The distinctness of the *D. byblica* species group has been demonstrated in several previous studies (Marchán et al. 2021b; Csuzdi et al. 2023; Shekhovtsov et al. 2024). The absence of calciferous diverticula and reddish pigmentation, and the broadly similar positions of the clitellum (approximately segments 25–30) and tubercles (approximately segments 26–28) render these Levantine, Anatolian, Central Asian, and Balkanic taxa relatively uniform.

Two peculiar species forming a clade are also recovered outside *Dendrobaena* sensu stricto in our analyses. One of these is *D. pantaleonis*, which is characterized by a ring-shaped clitellum extending across segments 24–29 and is distributed in France, Italy, Albania, Greece, Turkey, and Cyprus (Szederjesi 2017). The other species is *D. skipetarica*, an Albanian endemic, which in the study of Szederjesi et al. (2018) was recovered basal to the *D. byblica* species group together with *D. loebli* (Zicsi, 1985).

In summary, our study, which focused on the phylogenetic relationships within the genus *Dendrobaena* and several related genera, including *Eisenia*, involved a large number of species from a wide geographical area, and demonstrated the polyphyletic nature of both *Dendrobaena* and *Eisenia* genera. Our molecular phylogenetic results are well supported by morphological characters as well (Table 1); however, we are aware of the limitations of our research. These findings require improvement and corroboration with the inclusion of more representatives of the studied genera, and using a broader set of molecular markers or genomic approaches e.g. mitochondrial genomes or the Anchored hybrid enrichment (AHE) (Lemmon et al. 2012).

**Table 1.** The clades revealed and their main morphological characteristics.

**Clades**	**Setal arrangement**	**Calciferous diverticula**	**Last pair of hearts**	**Pigmentation**	**Musculature**
Red clade – “*Dendrobaena* s. str.”	Distant	11, 12	9 or 10, or less developed in 11	Dark red to unpigmented	Pinnate
Turquoise clade – “*Dendrodriloides*”	Closely paired to distant	Lacking	11 with extraoesophageals in 12	Red to unpigmented	Fasciculated
Blue clade – “*D. schmidti*”	Distant	12	11 with extraoesophageals in 12	Dark red to unpigmented	Pinnate
Orange clade – “*Fitzingeria*”	Distant	11	11 with extraoesophageals in 12	Dark red	Pinnate
Purple clade – “*D. pantaleonis*”	Distant	Lacking	11 with extraoesophageals in 12	Dark red to unpigmented	Pinnate
Grey clade – “*Omodeoia*”	Distant	Lacking	11 with extraoesophageals in 12	Pink to unpigmented	Pinnate
Green clade – “*Eisenia* s. str.”	Closely paired	11 and/or 12	11 with extraoesophageals in 12	Dark red to unpigmented	Pinnate, fasciculated or intermediate

## Additional information

### Artificial Intelligence (AI) use

The authors accept full responsibility for the content of the manuscript, including the disclosure of any use of AI.

No AI tools were used in the preparation of this manuscript.

### Author ORCIDs

T. Szederjesi https://orcid.org/0000-0001-7695-1468

T. Pavlíček https://orcid.org/0000-0003-2581-2657

A. Kliszcz https://orcid.org/0000-0002-1270-4414

C. Csuzdi https://orcid.org/0000-0002-0319-7836

### Data availability

All of the data that support the findings of this study are available in the main text or Supplementary Information.

## Supplementary materials

Supplementary material 1 Phylogenetic data Data type docx Explanation note List of species, localities, HNHM codes (if available) and GenBank accession numbers of the sequences with references used in the phylogenetic analyses, newly obtained sequences are in bold. https://binary.pensoft.net/file/1725013This dataset is made available under the Open Database License (http://opendatacommons.org/licenses/odbl/1.0/). The Open Database License (ODbL) is a license agreement intended to allow users to freely share, modify, and use this Dataset while maintaining this same freedom for others, provided that the original source and author(s) are credited. Tímea Szederjesi, Tomáš Pavlíček, Angelika Kliszcz, Csaba Csuzdi
