# Supplementary material for: Mission possible: An attempt toward a phylogenetic understanding of the genus Dendrobaena (Crassiclitellata, Lumbricidae)
Source: Zookeys. 2026 Jul 24;1286:105–19. doi: 10.3897/zookeys.1286.201330 (PMC13428245; doi:10.3897/zookeys.1286.201330)
Supplement: Supplementary material 1 — Phylogenetic data [file zookeys-1286-105_article-201330__-s001.docx]

List of species, localities, HNHM codes (if available) and GenBank accession numbers of the sequences with references used in the phylogenetic analyses, newly obtained sequences are in bold.

| **Species** | **Country** | **HNHM code** | **COI** | **16S rDNA** | **ITS2** | **Reference** |
| --- | --- | --- | --- | --- | --- | --- |
| *Ap. caliginosa* | Sweden |  | PP148048.1 | PP146776.1 | PP146009.1 |  |
| *Ap. longa* | Sweden |  | PP148119.1 | PP146828.1 | PP146040.1 |  |
| *B. tumidus* | USA | 16503 | KX651124.1 | KX651228.1 | KX651395.1 | Csuzdi et al. (2017) |
| *B. schwerti* | USA | 17158 | KX651138.1 | KX651241.1 | KX651405.1 | Csuzdi et al. (2017) |
| *B. rubidus* | Turkey | 15816 | KX651131.1 | KX651235.1 | KX651401.1 | Csuzdi et al. (2017) |
| *C. gestroides* | Hungary | 15566 | KX009565.1 | **PV705414** | **PV705445** | Szederjesi et al. (2016) |
| *C. sturanyi dacica* | Romania | 16737 | **PV707986** | **PV705412** | **PV705444** |  |
| *C. sturanyi dacidoides* | Romania | 15348 | KX009570.1 | **PV705413** | **PV715778** | Szederjesi et al. (2016) |
| *C. sturanyi sturanyi* | Croatia | 15842 | KX009567.1 | **PV705415** | **PV705446** | Szederjesi et al. (2016) |
| *Dendrobaena sp.* | Azerbaijan |  | PQ279309.1 | PQ279309.1 |  | Shekhovtsov et al. (2024) |
| *D. alpina* | Georgia |  | PP922152.1 | PP922152.1 |  | Shekhovtsov et al. (2024) |
| *D. alpina* | Azerbaijan |  | PQ279308.1 | PQ279308.1 |  | Shekhovtsov et al. (2024) |
| *D. alpina* | Austria | 16689 | MH476302.1 | MH476312.1 | MH469554.1 | Szederjesi et al. (2019) |
| *D. alpina* | Austria | 17294 | PP627489.1 | PP626738.1 | PP626716.1 | Szederjesi et al. (2025) |
| *D. alpina* | North Macedonia | 17495 | PP627490.1 | PP626739.1 | PP626715.1 | Szederjesi et al. (2025) |
| *D. alpina alteclitellata* | Romania | 16301 | PP627488.1 | PP626737.1 |  | Szederjesi et al. (2025) |
| *D. alpina alteclitellata* | Romania | 16454 | OM296013.1 | OM296300.1 | OM296312.1 | Csuzdi et al. (2023) |
| *D. annectens* | Romania | 16249 | KT823834.1 | KT823875.1 | KT823907.1 | Szederjesi et al. (2018) |
| *D. armeniaca* | Azerbaijan | 17552 | PP627475.1 | PP626725.1 | PP626723.1 | Szederjesi et al. (2025) |
| *D. attemsi* | Portugal | 16468 | KX651127.1 | KX651231.1 | KX651397.1 | Csuzdi et al. (2017) |
| *D. balcanica* | Greece | 15774 | PP627478.1 | PP626727.1 | PP626724.1 | Szederjesi et al. (2025) |
| *D. byblica* | Albania | 16160 | KT823828.1 | KT823869.1 | KT823901.1 | Szederjesi et al. (2018) |
| *D. byblica* | Greece | 16185 | KT823820.1 | KT823861.1 | KT823898.1 | Szederjesi et al. (2018) |
| *D. byblica* | Serbia | 16457 | KT823830.1 | KT823871.1 | KT823903.1 | Szederjesi et al. (2018) |
| *D. byblica* | Turkey | 16924 | KT823813.1 | KT823854.1 | **PV705447** | Szederjesi et al. (2018) |
| *D. carpathomontana* | Romania | 15963 | KT823845.1 | KT823886.1 | KT823914.1 | Szederjesi et al. (2018) |
| *D. clujensis* | Hungary | 16327 | OM296017.1 | OM296304.1 |  | Csuzdi et al. (2023) |
| *D. clujensis* | Romania | 16916 | PP627487.1 | PP626736.1 | PP626717.1 | Szederjesi et al. (2025) |
| *D. daghestanensis* | Georgia | 16105 | KT823826.1 | KT823867.1 | KT823900.1 | Szederjesi et al. (2018) |
| *D. daghestanensis* | Azerbaijan | 17567 | **PV707987** | **PV705416** | **PV705448** |  |
| *D. depressa* | Bulgaria | 16379 | KT823840.1 | KT823881.1 | KT823911.1 | Szederjesi et al. (2018) |
| *D. fridericae uludagi* | Turkey | 17101 | **PV707976** | **PV705401** |  |  |
| *D. ganglbaueri* | Croatia | 15595 | KT823838.1 | KT823879.1 | KT823909.1 | Szederjesi et al. (2018) |
| *D. ganglbaueri* | Hungary | 16415 | KT823839.1 | KT823880.1 | KT823910.1 | Szederjesi et al. (2018) |
| *D. herculis* | Romania | 16231 | KT823904.1 | KT823872.1 | KT823831.1 | Szederjesi et al. (2018) |
| *D. hortensis* | Azerbaijan |  | PQ015122.1 | PQ015122.1 |  | Shekhovtsov et al. (2024) |
| *D. hortensis* | Georgia |  | PQ279307.1 | PQ279307.1 |  | Shekhovtsov et al. (2024) |
| *D. hortensis* | Georgia |  | PP922157.1 | PP922157.1 |  | Shekhovtsov et al. (2024) |
| *D. hortensis* | Turkey | 15418 | MH476304.1 | MH476314.1 | MH469549.1 | Szederjesi et al. (2019) |
| *D. hrabei* | North Macedonia | 15936 | **PV707985** | **PV705411** |  |  |
| *D. hrabei* | Bulgaria | 16899 | **PV707977** | **PV705402** |  |  |
| *D. hrabei* | Bulgaria | 16900 | MH476305.1 | MH476315.1 | MH469550.1 | Szederjesi et al. (2019) |
| *D. karacadagi* | Turkey | 16590 | MH476310.1 | MH476320.1 |  | Szederjesi et al. (2019) |
| *D. karacadagi* | Turkey | 16859 | MH476311.1 | MH476321.1 | MH469547.1 | Szederjesi et al. (2019) |
| *D. misirlioglui* | Bulgaria | 16377 | OM296012.1 | OM296299.1 | OM296311.1 | Csuzdi et al. (2023) |
| *D. misirlioglui* | Bulgaria | 16388 | OM296018.1 | OM296305.1 |  | Csuzdi et al. (2023) |
| *D. nassonovi nassonovi* | Georgia |  | PP873346.1 | PP873346.1 |  | Shekhovtsov et al. (2024) |
| *D. octaeda* | Montenegro | 16212 | KX651128.1 | KX651232.1 | KX651398.1 | Csuzdi et al. (2017) |
| *D. octaeda* | France | 16528 | KX651156.1 | KX651233.1 | KX651399.1 | Csuzdi et al. (2017) |
| *D. olympiaca* | Greece | 15824 | KT823833.1 | KT823874.1 | KT823906.1 | Szederjesi et al. (2018) |
| *D. orientalis* | Israel | 16267 | MH476306.1 | MH476316.1 | MH469553.1 | Szederjesi et al. (2019) |
| *D. orientalis* | Israel | 16271 | OM296009.1 | OM296296.1 | OM296308.1 | Csuzdi et al. (2023) |
| *D. orientaloides* | Turkey | 16575 | OM296011.1 | OM296298.1 | OM296310.1 | Csuzdi et al. (2023) |
| *D. pantaleonis* | Greece | 17000 | MH476307.1 | MH476317.1 | MH469555.1 | Szederjesi et al. (2019) |
| *D. pantaleonis* | Greece | 17003 | **PV707988** | **PV705417** | **PV705449** |  |
| *D. pantaleonis* | Greece | 17023 | **PV707979** | **PV705404** |  |  |
| *D. pavliceki* | Turkey | 17082 | MH476308.1 | MH476318.1 |  | Szederjesi et al. (2019) |
| *D. pentheri* | Greece | 16184 | KT823844.1 | KT823885.1 | KT823913.1 | Szederjesi et al. (2018) |
| *D. pentheri* | Cyprus | 16515 | KT823846.1 | KT823887 | KT823915.1 | Szederjesi et al. (2018) |
| *D. pentheri* | Turkey | 16571 | **PV707980** | **PV705405** |  |  |
| *D. pentheri* | Turkey | 16572 | **PV707981** | **PV705406** | **PV705443** |  |
| *D. pentheri* | Turkey | 16923 | **PV707989** | **PV705418** | **PV705450** |  |
| *D. persimilis* | Turkey | 17052 | PP627477.1 | PP626726.1 | PP626721.1 | Szederjesi et al. (2025) |
| *D. persimilis* | Turkey | 17053 | **PV707982** | **PV705407** |  |  |
| *D. persimilis* | Turkey | 17078 | **PV707983** | **PV705408** |  |  |
| *D. platyura* | Hungary | 14547 | KT823849.1 | KT823890.1 | KT823918.1 | Szederjesi et al. (2018) |
| *D. platyura* | Hungary | 16439 | KT823847.1 | KT823888.1 | KT823916.1 | Szederjesi et al. (2018) |
| *D. popi* | Romania | 17203 | PP627479.1 | PP626728.1 | PP626720.1 | Szederjesi et al. (2025) |
| *D. popi* | Romania | 17219 | PP627480.1 | PP626729.1 | PP626719.1 | Szederjesi et al. (2025) |
| *D. ressli* | Turkey | 17057 | **PV707984** | **PV705409** |  |  |
| *D. schelkovnikovi bakuensis* | Azerbaijan | 17439 | **PV707990** | **PV705419** | **PV705451** |  |
| *D. schmidti* | Georgia |  | PP922156.1 | PP922156.1 |  | Shekhovtsov et al. (2024) |
| *D. schmidti* | Georgia |  | PP949297.1 | PP949297.1 |  | Shekhovtsov et al. (2024) |
| *D. schmidti* | Georgia |  | PP922154.1 | PP922154.1 |  | Shekhovtsov et al. (2024) |
| *D. semitica* | Turkey | 16279 | OM296010.1 | OM296297.1 | OM296309.1 | Csuzdi et al. (2023) |
| *D. semitica* | Israel | 16268 | PP627483.1 | PP626732.1 | PP626718.1 | Szederjesi et al. (2025) |
| *D. semitica* | Turkey | 16508 | **PV707991** | **PV705420** | **PV705452** |  |
| *D. semitica* | Cyprus | 16712 | MH476309.1 | MH476319.1 | MH469552.1 | Szederjesi et al. (2019) |
| *D. skipetarica* | Albania | 15864 | KT823818.1 | KT823859.1 | KT823897.1 | Szederjesi et al. (2018) |
| *D. succinta* | Israel | 16266 | MF417009.1 | MF417031.1 | MH469548.1 | Szederjesi et al. (2019) |
| *D. taurica* | Turkey | 17061 | - | **PV705410** |  |  |
| *D. transylvanica* | Romania | 15497 | KT823835.1 | KT823876.1 | KT823908.1 | Szederjesi et al. (2018) |
| *D. transylvanica* | Romania | 16490 | KT823837.1 | KT823878.1 | **PV705453** | Szederjesi et al. (2018) |
| *D. veneta* | Russia |  | PQ214936.1 | PQ214936.1 |  | Shekhovtsov et al. (2024) |
| *D. veneta* | Turkey | 16098 | KT823850.1 | KT823891.1 | KT823919.1 | Szederjesi et al. (2018) |
| *D. veneta* | Hungary | 16322 | KT823848.1 | KT823889.1 | KT823917.1 | Szederjesi et al. (2018) |
| *D. veneta* | Greece | 16619 | MF417020.1 | MF417042.1 | MH469546.1 | Szederjesi et al. (2019) |
| *Dd. grandis* | Armenia |  | PQ279313.1 | PQ279313.1 |  | Shekhovtsov et al. (2024) |
| *Dd. grandis* | Azerbaijan | 17525 | **PV707992** | **PV705421** |  |  |
| *Dd. perelae* | Georgia | 17595 | **PV707993** | **PV705422** |  |  |
| *Dd. supsaiensis* | Georgia | 17606 | **PV707994** | **PV705423** |  |  |
| *E. ebneri* | Greece | 15836 | KJ866404.1 | **PV705424** | **PV705454** | Szederjesi and Csuzdi (2015) |
| *E. ebneri* | Greece | 17020 | **PV707995** | **PV705425** |  |  |
| *E. ebneri* | Greece | 17024 | **PV707996** | **PV705426** |  |  |
| *E. fetida* |  |  | MF121770.1 | KJ912545.1 | JX531618.1 | Porco et al. (2018), Domínguez et al. (2015), Shekhovtsov et al. (2013) |
| *E. iverica* | Georgia | 17539 | **PV707997** | **PV705427** |  |  |
| *E. kontschani* | Turkey | 16954/1 | **PV707998** | **PV705428** | **PV705455** |  |
| *E. kontschani* | Turkey | 16954/2 | **PV707999** | **PV705429** | **PV705456** |  |
| *E. lagodechiensis* | Azerbaijan | 17571 | **PV708000** | **PV705430** |  |  |
| *E. muranyii* | Albania | 16117 | KJ866408.1 | **PV705431** |  | Szederjesi and Csuzdi (2015) |
| *E. muranyii* | Albania | 17590 | **PV708001** | **PV705432** |  |  |
| *E. muranyii* | Albania | 17615 | **PV708002** | **PV705433** |  |  |
| *E. nordenskioldi nordenskioldi* | Russia |  | MK618509.1 | MK618509.1 |  | Shekhovtsov et al. (2020) |
| *E. nordenskioldi pallida* | Russia |  | MK618512.1 | MK618512.1 |  | Shekhovtsov et al. (2020) |
| *E. oreophila* | Greece | 15912 | KJ866409.1 | **PV705434** |  | Szederjesi and Csuzdi (2015) |
| *E. oreophila* | Greece | 15912 | KJ866410.1 | **PV705435** | **PV705457** | Szederjesi and Csuzdi (2015) |
| *E. patriciae* | Turkey | 16592 | **PV708003** | **PV705436** |  |  |
| *E. spelaea* | Hungary |  | MK642870.1 | MK642870.1 |  | Shekhovtsov et al. (2020) |
| *E. storkani* | Bulgaria | 15334 | KJ866413.1 | **PV705437** | **PV705458** | Szederjesi and Csuzdi (2015) |
| *He. boluana* | Turkey | 17095 | **PV708004** | **PV705438** | **PV705459** |  |
| *He. mariae* | Turkey | 16939 | **PV708005** | **PV705439** | **PV705460** |  |
| *He. syriaca* | Israel | 16276 | **PV708006** | **PV705440** | **PV705461** |  |
| *He. zicsii* | Turkey | 17079 | **PV708007** | **PV705441** | **PV705462** |  |
| *H. oculatus* | Norway |  | MW535852.1 | MW544099.1 | MW534325.1 | Martinsson et al. (2021) |
| *H. oculatus* | Sweden |  | MW535870.1 | MW544087.1 | MW534322.1 | Martinsson et al. (2021) |
| *I. balcanicus plavensis* | Kosovo | 17426 | OL614457.1 | OL621919.1 | **PV705463** | Szederjesi et al. (2023) |
| *I. kratochvili* | Bosnia and Herzegovina | 16960 | OL614456.1 | OL621917.1 | **PV700408** | Szederjesi et al. (2023) |
| *I. movilensis* | Romania | 17667 | OL614462.1 | OL621921.1 | **PV715779** | Szederjesi et al. (2023) |
| *I. mozsaryorum* | Hungary | 16134 | KT823853.1 | KT823894.1 | KT8239221.1 | Szederjesi et al. (2018) |
| *I. patriarchalis* | Turkey | 16537 | **PV708008** | **PV705442** | **PV705464** |  |
| *I. vagneri* | Greece | 15725 | KT823851.1 | KT823892.1 | KT823920.1 | Szederjesi et al. (2018) |
| *O. cyaneum* | Norway |  | PP148766.1 | PP147358.1 | PP146367.1 |  |
| *O. lacteum* | Norway |  | PP148810.1 | PP147387.1 | PP146377.1 |  |
| *Ph. baloutchi* | Iran |  | Bozorgi *et al.* (2019) | LC427085.1 |  | Bozorgi et al. (2019) |
| *Pr. thaleri* | Romania | 16197 | OL614450.1 | OL621908.1 | **PV705465** | Szederjesi et al. (2023) |
| *Pr. tuberculatus* | Cyprus | 16514 | OL614453.1 | OL621913.1 | **PV705466** | Szederjesi et al. (2023) |
| *Sp. antiquus* | Greece | 15756 | KX651136.1 |  | KX651403.1 | Csuzdi et al. (2017) |

**References**

Bozorgi F, Seiedy M, Malek M, Aira M, Pérez-Losada M, Domínguez J (2019) Multigene phylogeny reveals a new Iranian earthworm genus (Lumbricidae: *Philomontanus*) with three new species. PLOS ONE 14(1): e0208904. <https://doi.org/10.1371/journal.pone.0208904>

Csuzdi Cs, Chang C-H, Pavlíček T, Szederjesi T, Esopi D, Szlávecz K (2017) Molecular phylogeny and systematics of native North American lumbricid earthworms (Clitellata: Megadrili). PLOS ONE 12(8): e0181504. <https://doi.org/10.1371/journal.pone.0181504>

Domínguez J, Aira M, Breinholt JW, Stojanović M, James SW, Pérez-Losada M (2015) Underground evolution: New roots for the old tree of lumbricid earthworms. Molecular Phylogenetics and Evolution 83: 7–19. <https://doi.org/10.1016/j.ympev.2014.10.024>

Porco D, Chang C-H, Dupont L, James S, Richard B, Decaëns T (2018) A reference library of DNA barcodes for the earthworms from Upper Normandy: biodiversity assessment, new records, potential cases of cryptic diversity and ongoing speciation. Applied Soil Ecology 124: 362–371. <https://doi.org/10.1016/j.apsoil.2017.11.001>

Shekhovtsov SV, Golovanova EV, Peltek SE (2013) Cryptic diversity within the Nordenskiold’s earthworm, *Eisenia nordenskioldi* subsp. *nordenskioldi* (Lumbricidae, Annelida). European Journal of Soil Biology 58: 13–18. <https://doi.org/10.1016/j.ejsobi.2013.05.004>

Szederjesi T, Pop VV, Márton O, Krízsik V, Csudi Cs (2016) The *Allolobophora sturanyi* species group revisited: Integrated taxonomy and new taxa (Clitellata: Megadrili). Opuscula Zoologica Budapest 47(1): 87–92. <https://doi.org/10.18348/opzool.2016.1.87>

Szederjesi T, Marchán DF, Csuzdi Cs, Sarbu SM, Pavlíček T, Krízsik V, Martin P, Domínguez J (2023) Three in one: molecular phylogeny of the genus *Helodrilus* (Crassiclitellata: Lumbricidae) with a description of two new genera and two new species. Zoological Journal of the Linnean Society 197(4): 899–908. <https://doi.org/10.1093/zoolinnean/zlac069>
